# Association between liver fibrosis and thrombotic or bleeding events in acute coronary syndrome patients

**DOI:** 10.1186/s12959-022-00441-8

**Published:** 2022-12-28

**Authors:** Yupeng Liu, Jingjing Song, Wenyao Wang, Kuo Zhang, Jie Yang, Jun Wen, Xiangbin Meng, Jun Gao, Jingjia Wang, Chunli Shao, Yi-Da Tang

**Affiliations:** 1 Department of Cardiology, Guangdong Cardiovascular Institute, Guangdong Provincial People’s Hospital, Guangdong Academy of Medical Sciences, Guangzhou, China; 2 Department of Cardiology, State Key Laboratory of Cardiovascular Disease, Fuwai Hospital, National Center for Cardiovascular Diseases, Chinese Academy of Medical Sciences and Peking Union Medical College, Beijing, China; 3 Department of Cardiology and Institute of Vascular Medicine, Peking University Third Hospital , Beijing, China; 4 Key Laboratory of Molecular Cardiovascular Science, Ministry of Education, Beijing, China

**Keywords:** Acute coronary syndrome, Liver fibrosis, Thrombotic events, Bleeding events, Risk stratification

## Abstract

**Background:**

The prognostic implication of liver fibrosis in acute coronary syndrome (ACS) patients are scarce. We sought to evaluate whether liver fibrosis scores (LFS) were associated with thrombotic or bleeding events in patients with acute coronary syndrome.

**Methods:**

We included 6386 ACS patients who underwent percutaneous coronary intervention (PCI). This study determined liver fibrosis with aspartate aminotransferase to platelet ratio index (APRI), aspartate aminotransferase to alanine aminotransferase ratio (AST/ALT ratio), Forns score, and nonalcoholic fatty liver disease fibrosis score (NFS). The primary endpoint was major adverse cardiac and cerebrovascular events (MACCE), a composite of all-cause mortality (ACM), myocardial infarction (MI), and ischemic stroke (IS).

**Results:**

During the follow-up, 259 (4.06%) MACCE and 190 (2.98%) bleeding events were recorded. As a continuous variable or a categorical variable stratified by the literature-based cutoff, LFS was positively associated with MACCE (*p* > 0.05) but not with bleeding events. Compared with subjects with low APRI scores, AST/ALT ratio scores, Forns scores, and NFS scores, subjects with high scores had a 1.57- to 3.73-fold increase risk of MACCE after adjustment (all *p* < 0.05). The positive relationship between LFS and MACCE was consistent in different subgroups.

**Conclusions:**

In ACS patients, increased LFS predicted an elevated risk of thrombotic events but not bleeding. LFS may contribute to thrombotic risk stratification after ACS.

**Supplementary Information:**

The online version contains supplementary material available at 10.1186/s12959-022-00441-8.

## Background

Acute coronary syndrome (ACS) is one of the leading causes of death worldwide [1]. Percutaneous coronary intervention (PCI), one of the most widely used therapeutic strategies for ACS patients, could improve patient outcomes. However, the risk of thrombotic and bleeding events after PCI remains high and may affect prognosis. The thrombotic and bleeding tradeoff is critical for the ACS patients. Therefore, exploring potential factors affecting thrombotic or bleeding events of ACS is meaningful in clinical management.

The global burden of chronic liver diseases is increasing rapidly. It is estimated that 1.5 billion people worldwide suffer from chronic liver diseases, and more than 2 million people die yearly [2]. Chronic injury in patients with chronic liver disease results in abnormal hepatic collagen increase and excessive extracellular matrix deposition in the liver, leading to liver fibrosis [3]. Liver fibrosis might silently exist in up to 9% of the general population [4]. Recent studies revealed that liver fibrosis was associated with coronary calcification and the severity of coronary artery diseases [5–7]. However, the relationship between liver fibrosis and adverse outcomes in ACS patients has been barely investigated.

The gold standard for liver fibrosis diagnosis is liver biopsy, an invasive procedure with a risk of complications [8]. High cost and sample variability also make liver biopsy unsuitable for routine population screening [9]. With the increasing burden of liver fibrosis and the need for simple evaluation tools, the liver fibrosis scores (LFS) calculated from clinical parameters and blood tests for predicting the severity of liver fibrosis were established and considered an alternative evaluation tool [10]. Compared with liver biopsy, LFS is simple, economical, non-invasive, and could be widely used in screening [11]. Commonly used LFS include the aspartate aminotransferase to platelet ratio index (APRI), Forns score, aspartate aminotransferase to alanine aminotransferase ratio (AST/ALT ratio) [12–14], and nonalcoholic fatty liver disease fibrosis score (NFS) [15]. In this study, we aimed to investigate whether LFS (e.g., APRI, AST/ALT ratio, Forns score, NFS) were associated with thrombotic and bleeding events in patients with ACS.

## Methods

### Study population

This study complied with the Declaration of Helsinki and was approved by the ethics committee of Fuwai Hospital. This study is an observational cohort study. Participants enrolled in this study have signed informed consent forms. From January to December 2013, a total of 10,724 patients who underwent PCI in Fuwai hospital were screened. The eligibility criteria are listed as the following: (1) ACS patients underwent PCI in Fuwai hospital, including patients with ST-segment elevation myocardial infarction (STEMI), non-ST-segment elevation myocardial infarction (NSTEMI), and patients with unstable angina (UA); (2) patients over 18 years old. The exclusion criteria are (1) patients with incomplete clinical information or incomplete follow-up; (2) patients lacking data on liver function, platelet count, and biochemical tests. Finally, 6386 ACS patients who underwent PCI in Fuwai hospital were enrolled in our study. Baseline demographic data, clinical data, and procedural data were collected prospectively. Before the procedure, patients were administered a loading dose of 300 mg aspirin and a loading dose of 300 mg clopidogrel or a loading dose of 180 mg ticagrelor. After the procedure, patients were administered lifelong aspirin and clopidogrel or ticagrelor for 12 months. Medical equipment use is at the cardiologist’s discretion based on the patient’s condition.

### Liver fibrosis scores

This study evaluated APRI, AST/ALT ratio, Forns score, and NFS. These scores were calculated as the following formula:

- APRI = AST (IU/L)/AST (the upper limit of normal, ULN) × 100 / platelet count (10^9/L)
- AST/ALT ratio = AST (IU/L)/ALT (IU/L)
- Forns score = 7.811–3.131 × log (platelet count [10^9/L]) + 0.781 × log (GGT [IU/L]) + 3.467 × log (age [year]) –0.014 × total cholesterol (mg/dL)
- NFS = − 1.675 + (0.037 × age [years]) + (0.094 * BMI [Kg/m2]) + (1.13 × diabetes [yes = 1, no = 0]) + (0.99 × AST/ALT ratio) – (0.013 × platelet count [109/L]) – (0.66 × albumin [g/dL])

Based on the cutoff values of previous literature, we divided LFS into low, intermediate, and high scores, indicative of mild, moderate, and advanced liver fibrosis. The cutoff values of APRI were 0.5 and 1.5 [12]. The cutoff values of Forns score were 4.2 and 6.9 [13]. The cutoff values of NFS were − 1.455 and 0.676 [15]. The cutoff values of the AST/ALT ratio were 0.8 and 2 [16, 17].

### Follow-up and endpoints

The patients were followed up after the procedure through telephone or outpatient clinics by professional staff for 30 days, 6 months, and every year after that. Major adverse cardiac and cerebrovascular events (MACCE), composed of all-cause death, myocardial infarction (MI), and ischemic stroke, were primary endpoint. The secondary endpoints were the components of MACCE separately, major bleeding, target vessel revascularization (TVR), and stent thrombosis. The definition of endpoints is according to the academic research consortium (ARC) and bleeding academic research consortium (BARC). All-cause death is caused by any causes. Cardiac death is caused by MI, congestive heart failure, malignant arrhythmia, or other direct cardiac causes. MI is defined according to the universal definition of myocardial infarction (UDMI). Ischemic stroke refers to the neurological dysfunction caused by ischemia of brain tissue and cells. Major bleeding refers to grade 2–5, based on the BARC definition [18].

### Statistical analysis

Continuous variables were reported as mean and standard deviation, and categorical variables as count and percentage. Continuous variables were compared by analysis of variance (ANOVA), and categorical variables were compared by the chi-square test. Unadjusted outcomes rates were estimated by Kaplan-Meier analysis and compared with the log-rank test. The Cox proportional hazards regression model was used to assess the association between LFS and clinical outcomes and recorded as hazard ratios (HR) and 95% confidence interval (CIs). Variables with *p* < 0.1 in univariable Cox regression analysis (Supplementary Table 1) and clinically relevant variables were used as adjusted variables. Variables included in the adjustment were shown in the following: age, sex, BMI, diabetes mellitus, hypertension, hyperlipidemia, smoking, previous PCI, previous cerebrovascular disease, left ventricular dysfunction (LVEF less than 50%), lesion number, and high-sensitive C reactive protein levels. Analytic components of LFS were not adjusted in the multivariable analysis. Restricted cubic splines were used to evaluate the correlation between continuous LFS and outcomes. Patients were divided into subgroups according to age (< 60 and ≥ 60), sex (male and female), BMI (< 28 and ≥ 28), smoking (yes or no), diabetes mellitus (yes or no), hypertension (yes or no), hyperlipidemia (yes or no) and STEMI (yes or no). We examined the consistency of the association between LFS and outcomes in different subgroups. Statistical analysis was performed using R 4.0.3 software. Two-tailed *p* < 0.05 was considered statistically significant.

## Results

### Baseline characteristics

6386 ACS patients who underwent PCI were enrolled in this study. The average age was 58.6 years old, and 4847(75.9%) were men. The baseline characteristics are presented according to the presence of liver fibrosis staged by APRI (cutoff 0.5 and 1.5) in Table 1. Compared to individuals with low fibrosis scores, those with intermediate and high fibrosis scores had a higher prevalence of male sex, STEMI, NSTEMI, left ventricular dysfunction (all *p* < 0.05). Moreover, compared with patients with low fibrosis scores, patients with intermediate and high fibrosis scores had significantly higher AST, ALT, total bilirubin, blood glucose and high-sensitivity C-reactive protein (all *p* < 0.05). The baseline characteristics of the study population by dividing into MACCE groups or non-MACCE groups were shown in Supplementary Table 2.

**Table 1:** Baseline characteristics according to the liver fibrosis status staged by APRI

Liver fibrosis	Low	Intermediate	High	***p*** Value
***Clinical characteristics***				
Age	58.7 (10.3)	57.6 (10.6)	58.1 (10.7)	0.028
Male	4235 (75.1)	542 (82.6)	70 (76.9)	< 0.001
BMI	25.9 (3.3)	25.7 (3.2)	26.1 (2.6)	0.29
Diabetes mellitus	1700 (30.1)	176 (26.8)	22 (24.2)	0.108
Hypertension	3680 (65.3)	390 (59.5)	55 (60.4)	0.009
Hyperlipidemia	3800 (67.4)	409 (62.3)	56 (61.5)	0.019
Smoking	3267 (57.9)	407 (62.0)	52 (57.1)	0.127
Prior PCI	1310 (23.2)	161 (24.5)	30 (33.0)	0.076
Prior CABG	225 (4.0)	24 (3.7)	4 (4.4)	0.898
Prior MI	772 (13.7)	84 (12.8)	9 (9.9)	0.485
Prior cerebrovascular disease	635 (11.3)	74 (11.3)	10 (11.0)	0.997
Peripheral vascular disease	154 (2.7)	18 (2.7)	2 (2.2)	0.953
STEMI	1139 (20.2)	277 (42.2)	58 (63.7)	< 0.001
NSTEMI	357 (6.3)	84 (12.8)	8 (8.8)	< 0.001
UA	4143 (73.5)	295 (45.0)	25 (27.5)	< 0.001
Left ventricular dysfunction	269 (4.8)	51 (7.8)	19 (20.9)	< 0.001
Renal insufficiency	2 (0.0)	2 (0.3)	0 (0.0)	0.032
***Procedural characteristics***				
Chronic total occlusion	396 (7.0)	24 (3.7)	2 (2.2)	0.001
Left main artery disease	333 (5.9)	37 (5.6)	5 (5.5)	0.952
Moderate to severe calcification	850 (15.1)	99 (15.1)	14 (15.4)	0.997
Lesion number	1.4 (0.7)	1.4 (0.7)	1.4 (0.7)	0.656
Lesion length	28.1 (18.8)	28.4 (17.2)	26.7 (17.1)	0.72
Minimum lesion diameter	0.4 (0.5)	0.3 (0.3)	0.2 (0.3)	< 0.001
Drug-eluting stent	5399 (95.7)	618 (94.2)	81 (89.0)	0.002
***Laboratory tests***				
AST, IU/L	20.0 (7.4)	56.1 (25.0)	216.9 (167.1)	< 0.001
ALT, IU/L	28.5 (19.3)	71.9 (55.6)	134.2 (142.8)	< 0.001
Albumin, g/dL	42.6 (4.2)	41.9 (4.7)	40.7 (4.2)	< 0.001
Total bilirubin, μmol/L	14.4 (5.4)	15.7 (6.9)	19.4 (10.5)	< 0.001
Triglycerides, mmol/L	1.8 (1.1)	1.8 (1.1)	1.8 (0.8)	0.499
Total cholesterol, mmol/L	4.2 (1.1)	4.2 (1.0)	4.5 (0.8)	0.043
LDL-C, mmol/L	2.5 (0.9)	2.5 (0.9)	2.7 (0.8)	0.075
HDL-C, mmol/L	1.0 (0.3)	1.0 (0.3)	1.1 (0.3)	0.04
Blood glucose, mmol/L	6.3 (2.3)	6.8 (3.0)	8.2 (4.5)	< 0.001
High-sensitivity C-reactive protein, mg/L	3.4 (3.9)	5.3 (5.0)	8.7 (5.8)	< 0.001

Variables are shown as mean (SD) or n (%). *BMI*, body mass index; *PCI*, percutaneous coronary intervention; *CABG*, coronary artery bypass grafting; *MI*, myocardial infarction; *STEMI*, ST-segment elevation myocardial infarction; *NSTEMI*, non-ST-segment elevation myocardial infarction *UA*, unstable angina; *ALT*, alanine aminotransferase; *AST*, aspartate aminotransferase; *LDL-C*, low-density lipoprotein cholesterol; *HDL-C*, high-density lipoprotein cholesterol

### Clinical outcomes according to liver fibrosis scores

The median follow-up time was 2.4 years. During the follow-up, 259 (4.06%) subjects had MACCE. 93 (1.46%) subjects had all-cause mortality, 50 (0.78%) subjects had cardiac death. 90 (1.41%) subjects had MI. 104 (1.63%) subjects had ischemic stroke. 190 (2.98%) subjects had major bleeding. 332 (5.2%) subjects had TVR. 66 (1.03%) subjects had stent thrombosis.

**Fig. 1:**
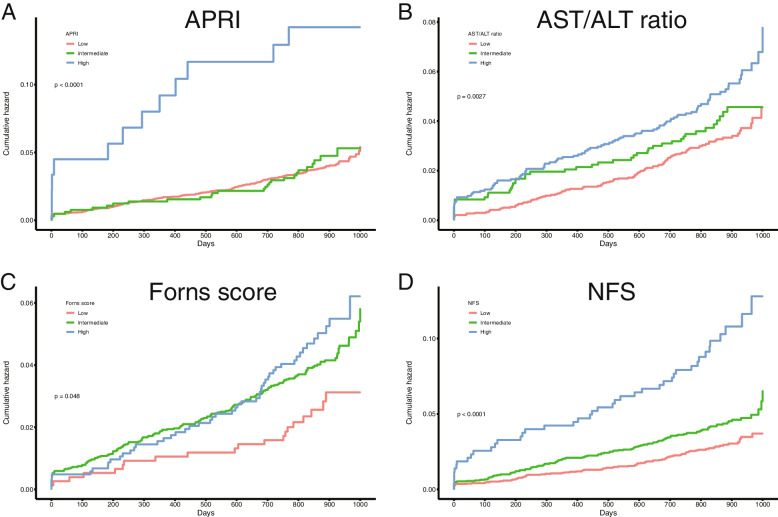
The Kaplan-Meier analysis for MACCE according to liver fibrosis scores. APRI, aspartate aminotransferase to platelet ratio index; AST/ALT ratio, aspartate aminotransferase to alanine aminotransferase ratio; NFS, nonalcoholic fatty liver disease fibrosis score

As shown in Fig. 1 and Table 2, increasing LFS was associated with progressively higher rates of MACCE. The incidence of MACCE increased from low LFS (2.6 to 3.9%) to high LFS (4.9 to 13.2%). Considering MACCE separately, mortality, myocardial infarction, ischemic stroke, and stent thrombosis had a positive relationship with fibrosis scores. All LFS was not associated with bleeding events and TVR (all *p* > 0.05) (Table 2).

**Table 2:** Clinical outcomes according to liver fibrosis status

Liver fibrosis staged by LFS	MACCE	All-cause death	Cardiac death	Myocardial infarction	Ischemic stroke	Major bleeding	TVR	Stent thrombosis
**APRI**								
Low (*N* = 5639)	219 (3.9)	77 (1.4)	37 (0.7)	72 (1.3)	89 (1.6)	169 (3.0)	289 (5.1)	53 (0.9)
Intermediate (*N* = 656)	28 (4.3)	10 (1.5)	7 (1.1)	14 (2.1)	11 (1.7)	18 (2.7)	38 (5.8)	8 (1.2)
High (*n* = 91)	12 (13.2)	6 (6.6)	6 (6.6)	4 (4.4)	4 (4.4)	3 (3.3)	5 (5.5)	5 (5.5)
*p* value	< 0.001	< 0.001	< 0.001	0.011	0.108	0.922	0.76	< 0.001
**AST/ALT ratio**								
Low (*N* = 3352)	112 (3.3)	31 (0.9)	18 (0.5)	37 (1.1)	54 (1.6)	94 (2.8)	182 (5.4)	30 (0.9)
Intermediate (*N* = 2666)	116 (4.4)	48 (1.8)	23 (0.9)	41 (1.5)	43 (1.6)	87 (3.3)	123 (4.6)	27 (1.0)
High (*N* = 368)	31 (8.4)	14 (3.8)	9 (2.4)	12 (3.3)	7 (1.9)	9 (2.4)	27 (7.3)	9 (2.4)
*p* value	< 0.001	< 0.001	< 0.001	0.003	0.913	0.481	0.06	0.02
**Forns score**								
Low (*N* = 763)	20 (2.6)	5 (0.7)	2 (0.3)	8 (1.0)	8 (1.0)	17 (2.2)	44 (5.8)	5 (0.7)
Intermediate (*N* = 4582)	188 (4.1)	58 (1.3)	32 (0.7)	66 (1.4)	83 (1.8)	135 (2.9)	242 (5.3)	45 (1.0)
High (*N* = 1041)	51 (4.9)	30 (2.9)	16 (1.5)	16 (1.5)	13 (1.2)	38 (3.7)	46 (4.4)	16 (1.5)
*p* value	0.051	< 0.001	0.005	0.648	0.174	0.209	0.397	0.152
**NFS**								
Low (*N* = 3109)	92 (3.0)	33 (1.1)	17 (0.5)	33 (1.1)	37 (1.2)	86 (2.8)	157 (5.0)	24 (0.8)
Intermediate (*N* = 2842)	124 (4.4)	41 (1.4)	20 (0.7)	43 (1.5)	53 (1.9)	89 (3.1)	150 (5.3)	28 (1.0)
High (*N* = 435)	43 (9.9)	19 (4.4)	13 (3.0)	14 (3.2)	14 (3.2)	15 (3.4)	25 (5.7)	14 (3.2)
*p* value	< 0.001	< 0.001	< 0.001	0.001	0.003	0.592	0.802	< 0.001

*LFS*, liver fibrosis scores; *MACCE*, major adverse cardiac and cerebrovascular events; *TVR*, target vessel reconstruction; *APRI*, aspartate aminotransferase to platelet ratio index; *AST/ALT ratio*, aspartate aminotransferase to alanine aminotransferase ratio; *NFS*, nonalcoholic fatty liver disease fibrosis score

### Association between liver fibrosis scores and MACCE

As displayed in Table 3, in unadjusted Cox regression analysis, subjects with high APRI scores exhibited an increased risk of MACCE compared to groups with low APRI scores (HR 3.69, 95%CI 2.06–6.6, *p* < 0.001). The MACCE risk increased in subjects with intermediate or high AST/ALT ratio (HR 1.31, 95%CI 1.01–1.69, *p* = 0.044; HR 2.59, 95%CI 1.74–3.85, *p* < 0.001), Forns score (HR 1.59, 95%CI 1.01–2.53, *p* = 0.047; HR 1.91, 95%CI 1.14–3.2, *p* = 0.015) and NFS (HR 1.48, 95%CI 1.13–1.94, *p* = 0.004; HR 3.43, 95%CI 2.39–4.92, *p* < 0.001) compared to the subjects with low scores. After adjustment for known predictors for MACCE (Table 4**)**, the MACCE risk still increased in patients with a high APRI (HR 3.73, 95%CI 2.03–6.87, *p* < 0.001), high NFS (HR 2.73, 95%CI 1.86–4, *p* < 0.001), high AST/ALT ratio (HR 1.57, 95%CI 1.02–2.43, *p* = 0.042) and high Forns score (HR 1.77, 95%CI 1.05–3, *p* = 0.032).

**Table 3:** Unadjusted Cox regression for clinical outcomes according to liver fibrosis status

Liver fibrosis staged by LFS	MACCE	All-cause death	Cardiac death	Myocardial infarction	Ischemic stroke	Major bleeding	TVR	Stent thrombosis
**APRI**								
Low (N = 5639)	1 (reference)	1 (reference)	1 (reference)	1 (reference)	1 (reference)	1 (reference)	1 (reference)	1 (reference)
Intermediate (N = 656)	1.1 (0.74–1.63)	1.12 (0.58–2.16)	1.62 (0.72–3.63)	1.67 (0.94–2.96)	1.06 (0.57–1.98)	0.91 (0.56–1.49)	1.13 (0.81–1.59)	1.29 (0.61–2.72)
*p* value	0.638	0.743	0.242	0.079	0.855	0.717	0.471	0.498
High (n = 91)	3.69 (2.06–6.6)	5.14 (2.24–11.8)	10.59 (4.47–25.09)	3.65 (1.33–9.99)	3.05 (1.12–8.3)	1.16 (0.37–3.65)	1.13 (0.47–2.74)	6.24 (2.49–15.61)
*p* value	< 0.001	< 0.001	< 0.001	0.012	0.029	0.794	0.784	< 0.001
**AST/ALT ratio**								
Low (N = 3352)	1 (reference)	1 (reference)	1 (reference)	1 (reference)	1 (reference)	1 (reference)	1 (reference)	1 (reference)
Intermediate (N = 2666)	1.31 (1.01–1.69)	1.94 (1.24–3.05)	1.61 (0.87–2.98)	1.4 (0.9–2.18)	1 (0.67–1.49)	1.17 (0.88–1.57)	0.85 (0.68–1.07)	1.13 (0.67–1.9)
*p* value	0.044	0.004	0.132	0.141	0.995	0.288	0.161	0.641
High (N = 368)	2.59 (1.74–3.85)	4.15 (2.21–7.8)	4.59 (2.06–10.21)	2.99 (1.56–5.74)	1.2 (0.55–2.64)	0.89 (0.45–1.75)	1.39 (0.93–2.08)	2.75 (1.31–5.8)
*p* value	< 0.001	< 0.001	< 0.001	0.001	0.647	0.726	0.11	0.008
**Forns score**								
Low (N = 763)	1 (reference)	1 (reference)	1 (reference)	1 (reference)	1 (reference)	1 (reference)	1 (reference)	1 (reference)
Intermediate (N = 4582)	1.59 (1.01–2.53)	1.95 (0.78–4.87)	2.69 (0.64–11.21)	1.39 (0.67–2.9)	1.76 (0.85–3.64)	1.34 (0.81–2.22)	0.92 (0.67–1.27)	1.52 (0.6–3.82)
*p* value	0.047	0.151	0.175	0.377	0.126	0.257	0.628	0.376
High (N = 1041)	1.91 (1.14–3.2)	4.46 (1.73–11.5)	5.94 (1.37–25.83)	1.49 (0.64–3.47)	1.22 (0.51–2.95)	1.68 (0.95–2.97)	0.77 (0.51–1.16)	2.39 (0.87–6.51)
*p* value	0.015	0.002	0.018	0.36	0.655	0.077	0.214	0.09
**NFS**								
Low (N = 3109)	1 (reference)	1 (reference)	1 (reference)	1 (reference)	1 (reference)	1 (reference)	1 (reference)	1 (reference)
Intermediate (N = 2842)	1.48 (1.13–1.94)	1.36 (0.86–2.15)	1.29 (0.67–2.46)	1.43 (0.91–2.25)	1.58 (1.04–2.4)	1.14 (0.84–1.53)	1.05 (0.84–1.31)	1.28 (0.74–2.21)
*p* value	0.004	0.192	0.445	0.124	0.034	0.401	0.688	0.377
High (N = 435)	3.43 (2.39–4.92)	4.14 (2.35–7.28)	5.5 (2.67–11.32)	3.06 (1.64–5.72)	2.75 (1.49–5.08)	1.27 (0.73–2.2)	1.16 (0.76–1.77)	4.2 (2.17–8.12)
*p* value	< 0.001	< 0.001	< 0.001	< 0.001	0.001	0.392	0.495	< 0.001

*LFS*, liver fibrosis scores; *MACCE*, major adverse cardiac and cerebrovascular events; *TVR*, target vessel reconstruction; *APRI*, aspartate aminotransferase to platelet ratio index; *AST/ALT ratio*, aspartate aminotransferase to alanine aminotransferase ratio; *NFS*, nonalcoholic fatty liver disease fibrosis score

**Table 4:** Adjusted Cox regression for clinical outcomes according to liver fibrosis status

Liver fibrosis staged by LFS	MACCE	All-cause death	Cardiac death	Myocardial infarction	Ischemic stroke	Major bleeding	TVR	Stent thrombosis
**APRI**								
Low (N = 5639)	1 (reference)	1 (reference)	1 (reference)	1 (reference)	1 (reference)	1 (reference)	1 (reference)	1 (reference)
Intermediate (N = 656)	1.14 (0.76–1.7)	0.97 (0.5–1.9)	1.43 (0.63–3.27)	1.91 (1.07–3.43)	1.15 (0.61–2.16)	0.99 (0.61–1.62)	1.13 (0.80–1.59)	1.35 (0.63–2.87)
*p* value	0.524	0.931	0.394	0.029	0.676	0.977	0.495	0.438
High (n = 91)	3.73 (2.03–6.87)	4.03 (1.66–9.78)	8.87 (3.32–23.72)	5.05 (1.76–14.5)	3.32 (1.17–9.43)	1.4 (0.44–4.45)	1.08 (0.44–2.66)	8.87 (3.29–23.94)
*p* value	< 0.001	0.002	< 0.001	0.003	0.024	0.572	0.862	< 0.001
**AST/ALT ratio**								
Low (N = 3352)	1 (reference)	1 (reference)	1 (reference)	1 (reference)	1 (reference)	1 (reference)	1 (reference)	1 (reference)
Intermediate (N = 2666)	0.92 (0.7–1.22)	1.13 (0.71–1.82)	0.92 (0.48–1.75)	1.14 (0.71–1.82)	0.69 (0.45–1.05)	0.89 (0.65–1.21)	0.87 (0.68–1.1)	0.99 (0.58–1.72)
*p* value	0.57	0.605	0.788	0.581	0.085	0.458	0.243	0.985
High (N = 368)	1.57 (1.02–2.43)	1.56 (0.77–3.13)	1.54 (0.62–3.8)	2.64 (1.29–5.41)	0.7 (0.3–1.62)	0.67 (0.33–1.37)	1.39 (0.90–2.14)	2.46 (1.06–5.67)
*p* value	0.042	0.214	0.352	0.008	0.408	0.276	0.141	0.035
**Forns score**								
Low (N = 763)	1 (reference)	1 (reference)	1 (reference)	1 (reference)	1 (reference)	1 (reference)	1 (reference)	1 (reference)
Intermediate (N = 4582)	1.55 (0.98–2.47)	2.1 (0.84–5.27)	2.89 (0.69–12.16)	1.26 (0.6–2.64)	1.62 (0.78–3.36)	1.26 (0.76–2.09)	0.91 (0.66–1.26)	1.36 (0.54–3.45)
*p* value	0.063	0.112	0.147	0.542	0.196	0.377	0.573	0.518
High (N = 1041)	1.77 (1.05–3)	4.46 (1.71–11.62)	5.59 (1.26–24.75)	1.32 (0.56–3.13)	1.08 (0.44–2.62)	1.57 (0.88–2.8)	0.74 (0.49–1.13)	1.88 (0.68–5.22)
*p* value	0.032	0.002	0.023	0.53	0.871	0.128	0.166	0.226
**NFS**								
Low (N = 3109)	1 (reference)	1 (reference)	1 (reference)	1 (reference)	1 (reference)	1 (reference)	1 (reference)	1 (reference)
Intermediate (N = 2842)	1.31 (1–1.72)	1.2 (0.76–1.92)	1.05 (0.55–2.03)	1.24 (0.78–1.96)	1.35 (0.88–2.07)	1.06 (0.78–1.43)	1.01 (0.81–1.27)	1.12 (0.65–1.96)
*p* value	0.052	0.434	0.875	0.364	0.168	0.702	0.928	0.679
High (N = 435)	2.73 (1.86–4)	2.77 (1.51–5.07)	3.49 (1.61–7.57)	2.77 (1.44–5.31)	2.12 (1.12–4.04)	1.24 (0.7–2.17)	1.08 (0.70–1.67)	4.08 (2.05–8.15)
*p* value	< 0.001	0.001	0.002	0.002	0.022	0.462	0.719	< 0.001

*LFS*, liver fibrosis scores; *MACCE*, major adverse cardiac and cerebrovascular events; *TVR*, target vessel reconstruction; *APRI*, aspartate aminotransferase to platelet ratio index; *AST/ALT ratio*, aspartate aminotransferase to alanine aminotransferase ratio; *NFS*, nonalcoholic fatty liver disease fibrosis score

To simply compare the outcomes between patients with liver cirrhosis and without, the whole population was divided into two groups with or without liver cirrhosis based on APRI (APRI cutoff at 1.5). We found that liver cirrhosis is associated with an increased risk of MACCE, all-cause death, cardiac death, MI, ischemic stroke, and stent thrombosis but not TVR and major bleeding (Table 5).

**Table 5:** Unadjusted and adjusted Cox regression for clinical outcomes according to liver cirrhosis evaluated by APRI

	Non-cirrhosis	Cirrhosis	***p*** value
***Unadjusted risk***			
MACCE	1(reference)	3.65 (2.05–6.52)	< 0.001
All-cause death	1(reference)	5.08 (2.22–11.62)	< 0.001
Cardiac death	1(reference)	9.95 (4.24–23.34)	< 0.001
MI	1(reference)	3.41 (1.25–9.30)	0.017
Ischemic stroke	1(reference)	3.03 (1.11–8.23)	0.030
TVR	1(reference)	1.12 (0.46–2.70)	0.81
Major bleeding	1(reference)	1.18 (0.38–3.68)	0.782
Stent thrombosis	1(reference)	6.06 (2.43–15.07)	< 0.001
***Adjusted risk***			
MACCE	1(reference)	3.65 (1.99–6.69)	< 0.001
All-cause death	1(reference)	4.05 (1.68–9.75)	0.002
Cardiac death	1(reference)	8.19 (3.11–21.53)	< 0.001
MI	1(reference)	4.45 (1.55–12.73)	0.005
Ischemic stroke	1(reference)	3.24 (1.15–9.17)	0.026
TVR	1(reference)	1.06 (0.43–2.60)	0.90
Major bleeding	1(reference)	1.40 (0.44–4.45)	0.57
Stent thrombosis	1(reference)	8.39 (3.14–22.47)	< 0.001

*MACCE*, major adverse cardiac and cerebrovascular event; *MI*, myocardial infarction; *TVR*, target vessel reconstruction. Values are hazard ratio (95% confidence interval)

**Fig. 2:**
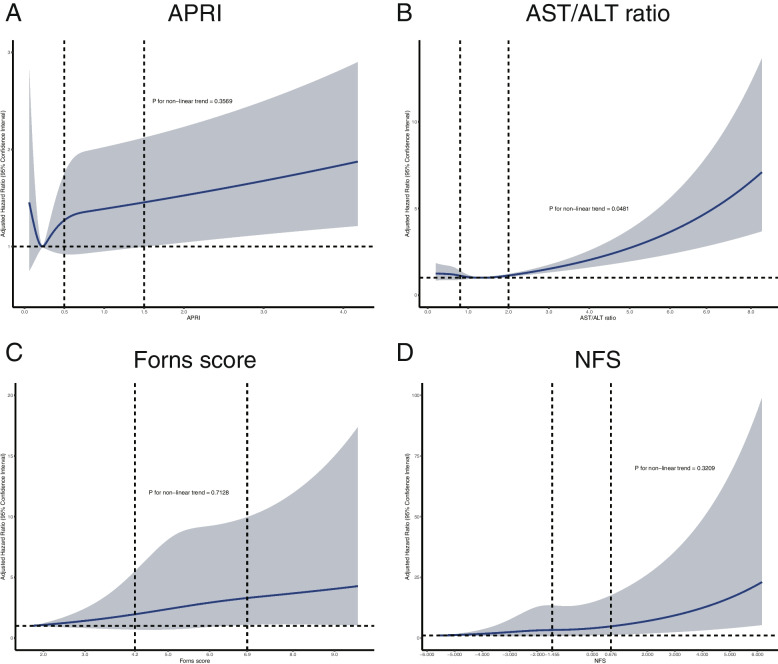
Restricted cubic spline analysis for MACCE according to liver fibrosis scores. APRI, aspartate aminotransferase to platelet ratio index; AST/ALT ratio, aspartate aminotransferase to alanine aminotransferase ratio; NFS, nonalcoholic fatty liver disease fibrosis score

As shown in Fig. 2, further adjusted restricted cubic spline analysis showed that as continuous variables, APRI, AST/ALT ratio, Forns score, and NFS were independently associated with increased MACCE risk. AST/ALT ratio had a non-linear positive relationship with MACCE risk, while APRI, NFS, and Forns score had a linear positive relationship with the MACCE risk.

As shown in Supplementary Fig. 1**–**4, patients were divided into subgroups according to age (< 60 and ≥ 60), sex (male and female), BMI (< 28 and ≥ 28), smoking (yes or no), diabetes mellitus (yes or no), hypertension (yes or no), hyperlipidemia (yes or no) and STEMI (yes or no). Consistently, patients with intermediate and high APRI, AST/ALT ratio, Forns score, and NFS tended to have an elevated risk of MACCE compared to those with a low score. This positive correlation was consistent in different subgroups.

### Association between liver fibrosis scores and secondary outcomes

Before and after adjustment, the risk of major bleeding did not significantly increase in groups with intermediate or high LFS (Table 3 and Table 4). Considering MACCE separately, elevated MACCE risks in the high APRI and NFS groups were mainly driven by increased mortality, MI, ischemic stroke and stent thrombosis (all *p* < 0.05). Elevated MACCE risks in the high Forns score group were primarily driven by increased mortality (*p* < 0.05). Elevated MACCE risks in the high AST/ALT ratio group were mainly driven by increased MI, and stent thrombosis (*p* < 0.05).

## Discussion

Previous studies demonstrated that LFS was associated with cardiovascular events [19–21]. Investigating the association of noninvasive fibrosis scores with thrombotic and bleeding events in acute coronary syndrome may open a new mind to thrombotic and bleeding tradeoffs in ACS management. Our study firstly evaluated the prognostic implication of APRI, AST/ALT ratio, Forns score, and NFS on the thrombotic and bleeding events of ACS patients who underwent PCI. We found that increased APRI, NFS, AST/ALT ratio, and Forns scores were associated with an elevated risk of thrombotic events but had no significant association with major bleeding. Compared to the patients with a low LFS, patients with a high LFS exhibited an increased risk of MACCE by 1.57- to 3.73-fold. Subgroup analysis showed that this positive correlation was consistent in different ages, sex, BMI, smoking status, diabetes mellitus, hypertension, hyperlipidemia, and STEMI subgroups.

Liver fibrosis severity indicates liver health status. Since the liver plays a critical role in lipid and glucose metabolism, and liver disease shares common risk factors with cardiovascular diseases, such as hypertension, insulin resistance, and systemic inflammation [22–25], it is reasonable to explore the relationship between liver fibrosis and cardiovascular diseases. As an alternative tool for liver biopsy, noninvasive LFS could evaluate liver fibrosis accurately and have good prognostic values for adverse outcomes. Elevated APRI and NFS were associated with an increased risk of mortality and hepatic complications in the NAFLD population [26]. National Health and Nutrition Examination Survey showed that increased APRI, Forns score, and NFS, were associated with increased risks of all-cause death, cardiac death, and liver-specific death in the U.S. population [27, 28]. NFS was associated with the risk of cardiovascular events in patients with diabetes mellitus [29]. These studies demonstrated that LFS was associated with hepatic and non-hepatic events in patients with and without liver diseases. Recent studies started exploring the relationship between LFS and cardiovascular disease. APRI and Forns scores were reported to be associated with mortality in intracerebral hemorrhage patients and ischemic stroke patients, respectively [30, 31]. Besides, APRI, Forns score, and NFS predicted all-cause mortality and cardiac death risks in coronary heart disease patients [28]. APRI was related to coronary calcification and severity in patients with coronary heart disease [5, 32]. Elevated AST/ALT ratio and NFS were associated with the increased risk of cardiovascular events in patients with the stable coronary disease after PCI [7]. This study is the first one exploring the relationship between LFS and thrombotic/bleeding events in ACS patients.

In our study, compared to the low score group, individuals with high APRI, AST/SLT ratio, Forns score, and NFS had a higher risk of MACCE. Additionally, APRI and NFS showed better performance predicting secondary outcomes than other scores, suggesting LFS could predict the risk of adverse outcomes in ACS patients undergoing PCI, which is consistent with previous studies. Besides, previous researches mainly focus on the predictive value of APRI or NFS on cardiovascular diseases. In our study, we comprehensively investigated four LFS, including APRI, AST/ALT ratio, Forns score, and NFS, to evaluate and compare their predictive values for thrombotic and bleeding events. And we found that LFS could predict thrombotic events in ACS patients but not bleeding events. Procoagulant imbalance is common in people with chronic liver disease, even those with mildly elevated liver fibrosis scores [33]. Inflammatory status, elevated thrombophilic factors, endothelial dysfunction, and oxidative injury in liver fibrosis or cirrhosis lead to hypercoagulable and thrombotic state [34, 35], increasing ischemic events risk. Besides, hyperlipidemia, insulin resistance, stress, and obesity, which are prevalent in ACS patients, may also exacerbate the hypercoagulable state [36]. This may be why liver fibrosis is associated with thrombotic events in our study but not bleeding. The ischemic and bleeding trade-off for ACS patients after PCI is vital. Our study suggested liver fibrosis staged by LFS was associated with ischemic events in ACS patients, and LFS might be a helpful tool for identifying patients with a high risk of ischemic events after PCI.

## Limitation

There are some limitations existing in our study. First, this study is an observational cohort study, which cannot identify a direct causal relationship because of the inherent limitations of such studies. Second, all patients received pre-loading of ticagrelor and or clopidogrel as recommended by the guidelines, which could influence the bleeding events in these populations. But we believed the treatment would not negate the bleeding characteristic of patients with high bleeding risk, which might require further study. Third, this study lacks liver biopsy or imaging techniques such as ultrasound elastography to confirm the severity of liver fibrosis. Recent studies suggested that the combination of imaging techniques and liver fibrosis scores could better diagnose liver fibrosis. And at the basis of this study, future research could seek to combine imaging techniques and LFS. Fourth, clopidogrel could cause thrombocytopenia, but this is a small probability event during PCI, according to previous reports. Fifth, since liver fibrosis scores could properly evaluate fibrosis in a different population, the present study focused on the extent of overall liver fibrosis. Additional information about the study population’s chronic liver disease status (e.g., viral hepatitis, NAFLD) was not collected, which might require further research. 

## Conclusions

This study found that elevate LFS was associated with an increased risk of MACCE, morality, MI, and stent thrombosis in patients with ACS, but not bleeding events and TVR. LFS may contribute to thrombotic risk stratification among ACS patients and help clinical decision making.

## Supplementary Information

**Additional file 1:** **Supplementary Fig. 1.** Subgroup analysis for APRI. APRI, aspartate aminotransferase to platelet ratio index.

**Additional file 2:** **Supplementary Fig. 2.** Subgroup analysis for AST/ALT ratio. AST/ALT ratio, aspartate aminotransferase to alanine aminotransferase ratio

**Additional file 3:** **Supplementary Fig. 3.** Subgroup analysis for Forns score

**Additional file 4:** **Supplementary Fig. 4.** Subgroup analysis for NFS. NFS, nonalcoholic fatty liver disease fibrosis score

**Additional file 5:** **Supplementary Table 1.** Univariable and multivariable Cox regression analysis of MACCE. **Supplementary Table 2**. Baseline characteristics according to the absence and presence of MACCE

## Data Availability

The data are not publicly available because of institutional review board restrictions.

## Abbreviations

LFS: liver fibrosis scores
APRI: aspartate aminotransferase to platelet ratio index
AST/ALT ratio: aspartate aminotransferase to alanine aminotransferase ratio
NFS: nonalcoholic fatty liver disease fibrosis score
ACS: acute coronary syndrome
PCI: percutaneous coronary intervention
MI: myocardial infarction
ALT: alanine aminotransferase
AST: aspartate aminotransferase
MACCE: major adverse cardiac and cerebrovascular events.
