# Supplementary figures and images for: Association between liver fibrosis and thrombotic or bleeding events in acute coronary syndrome patients
Source: Thromb J. 2022 Dec 28;20:82. doi: 10.1186/s12959-022-00441-8 (PMC9798679; doi:10.1186/s12959-022-00441-8)

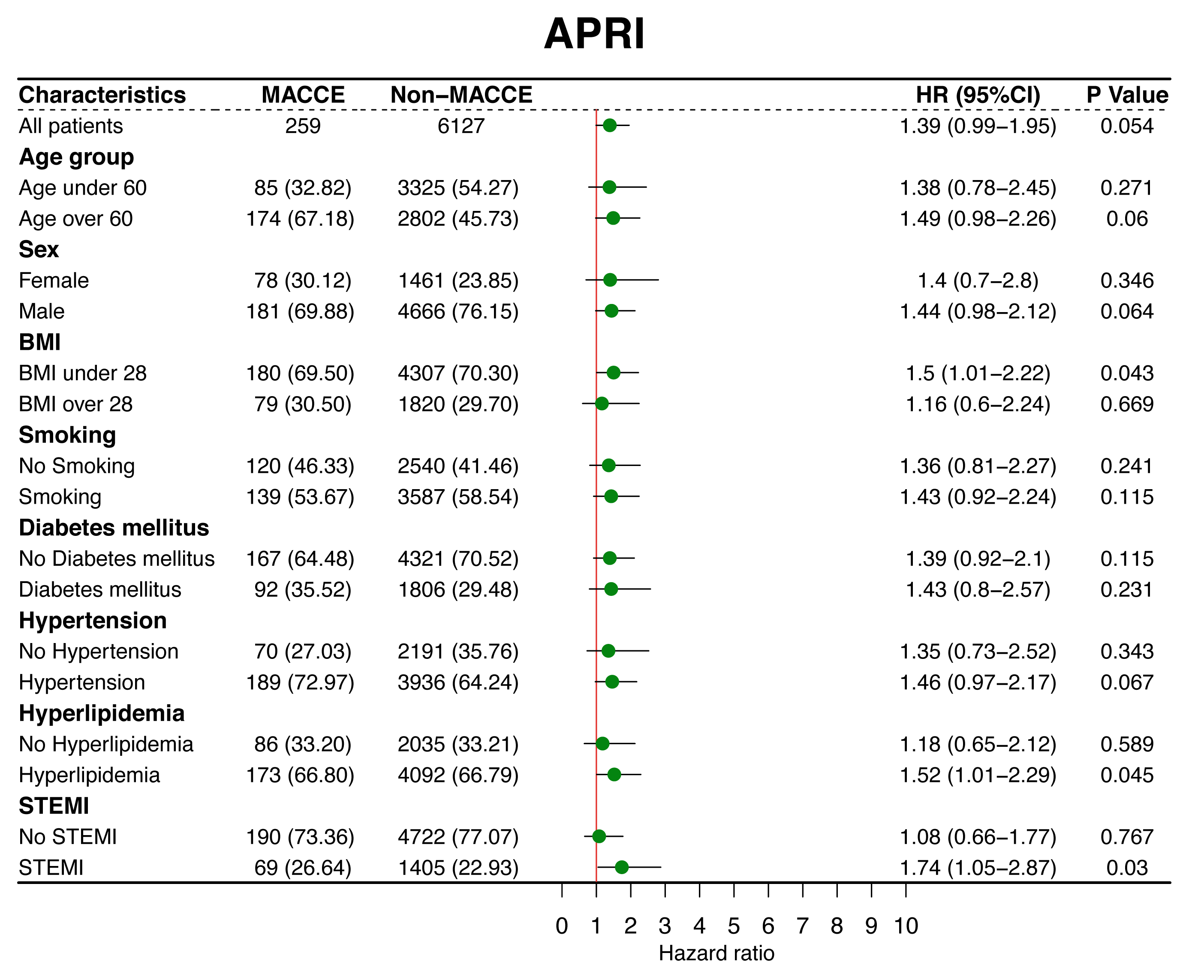

Supplement: Supplementary file 1 — Additional file 1: Supplementary Fig. 1. Subgroup analysis for APRI. APRI, aspartate aminotransferase to platelet ratio index. [file 12959_2022_441_MOESM1_ESM.tif]

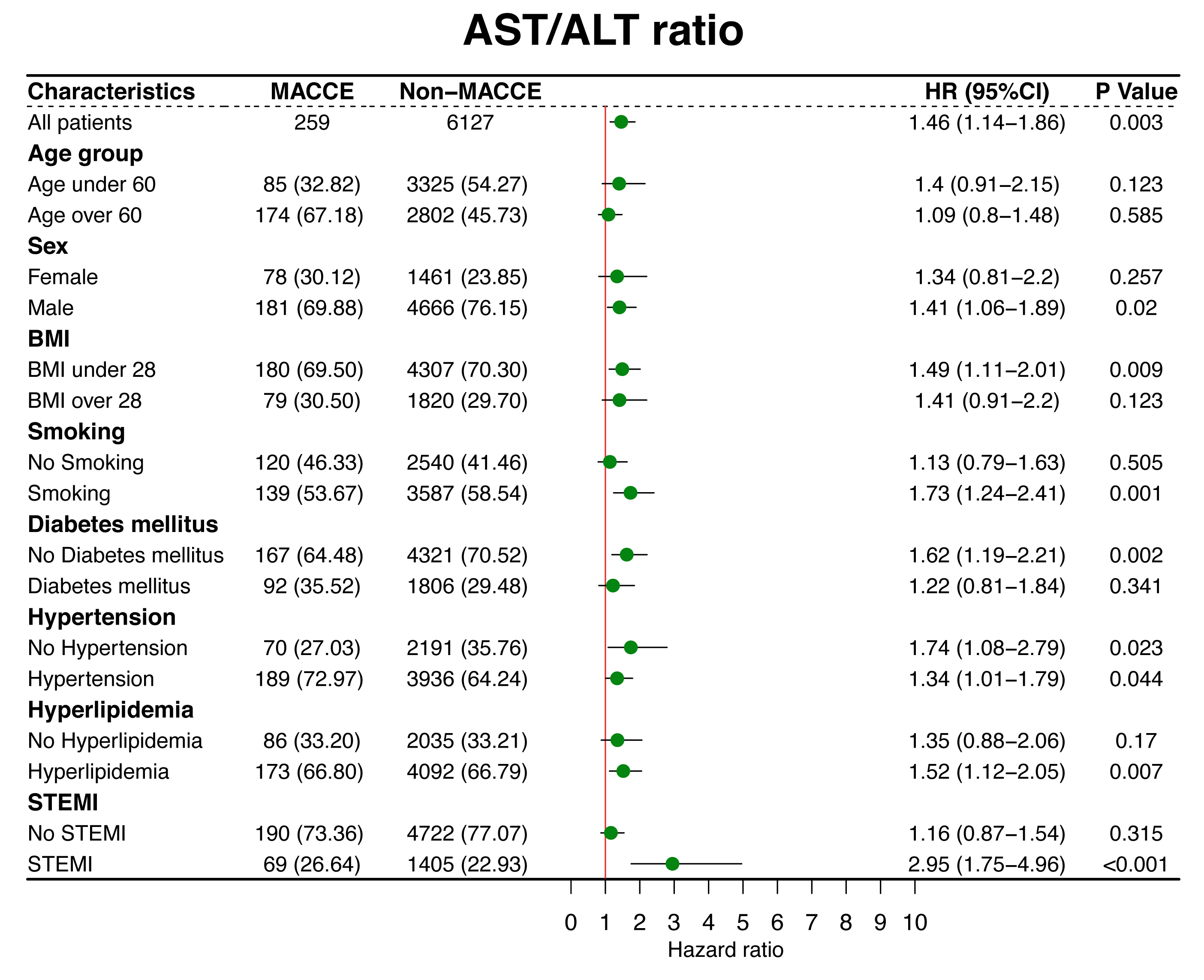

Supplement: Supplementary file 2 — Additional file 2: Supplementary Fig. 2. Subgroup analysis for AST/ALT ratio. AST/ALT ratio, aspartate aminotransferase to alanine aminotransferase ratio [file 12959_2022_441_MOESM2_ESM.tif]

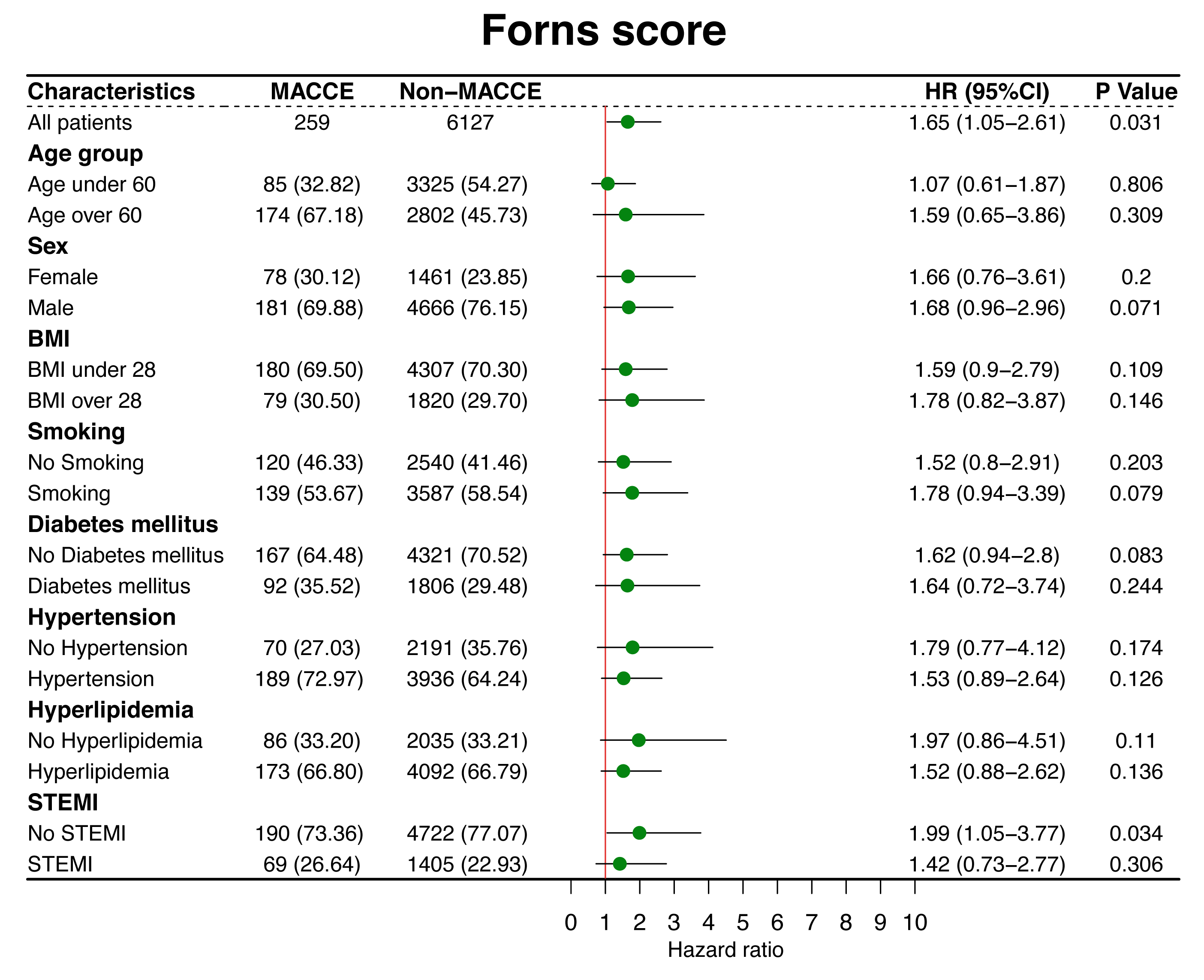

Supplement: Supplementary file 3 — Additional file 3: Supplementary Fig. 3. Subgroup analysis for Forns score [file 12959_2022_441_MOESM3_ESM.tif]

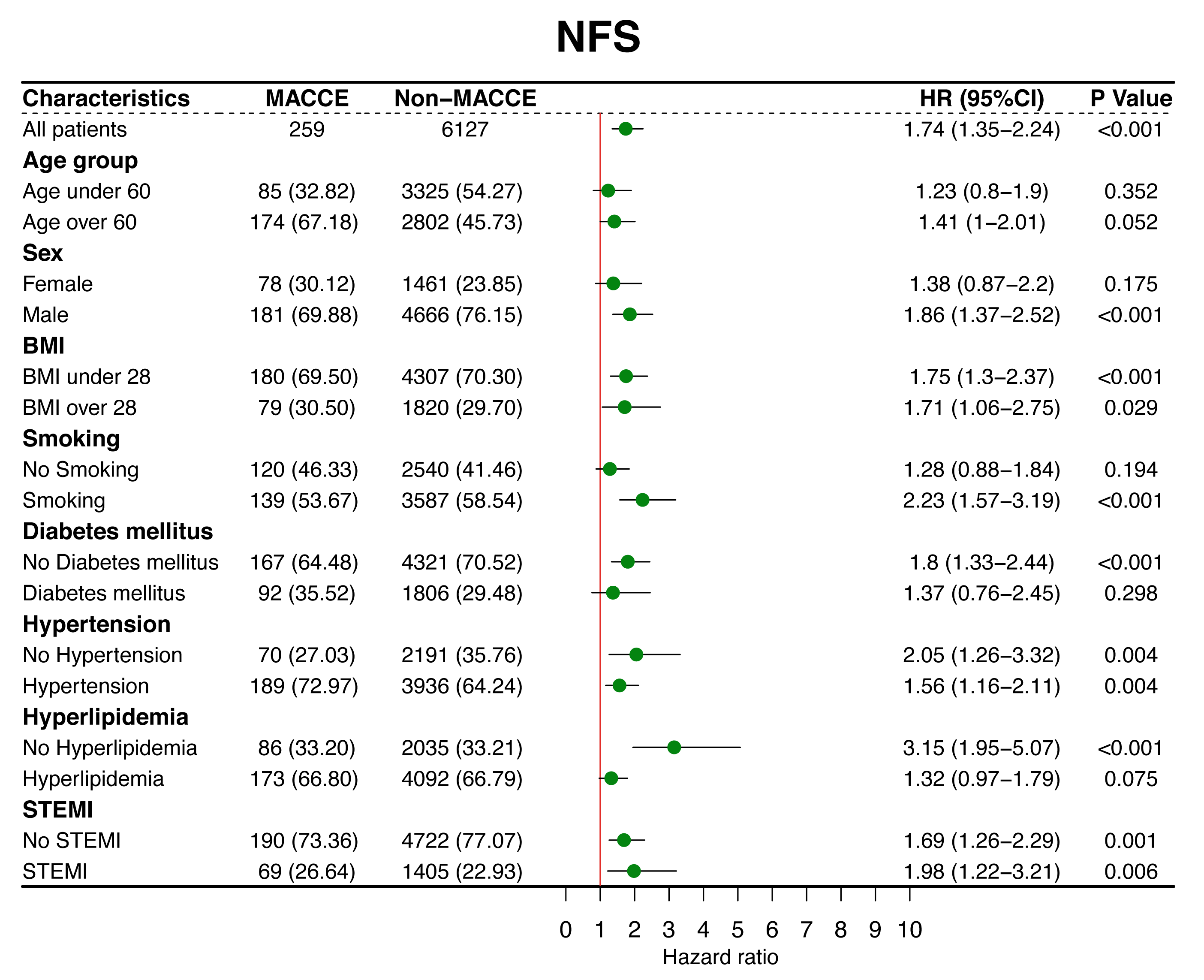

Supplement: Supplementary file 4 — Additional file 4: Supplementary Fig. 4. Subgroup analysis for NFS. NFS, nonalcoholic fatty liver disease fibrosis score [file 12959_2022_441_MOESM4_ESM.tif]
