# Supplementary material for: Association between liver fibrosis and thrombotic or bleeding events in acute coronary syndrome patients
Source: Thromb J. 2022 Dec 28;20:82. doi: 10.1186/s12959-022-00441-8 (PMC9798679; doi:10.1186/s12959-022-00441-8)
Supplement: Supplementary file 5 — Additional file 5: Supplementary Table 1. Univariable and multivariable Cox regression analysis of MACCE. Supplementary Table 2. Baseline characteristics according to the absence and presence of MACCE [file 12959_2022_441_MOESM5_ESM.docx]

**Supplementary Table 1. Univariable and multivariable Cox regression analysis of MACCE.**

|  | Univariable analysis | | | Multivariable analysis | | |
| --- | --- | --- | --- | --- | --- | --- |
|  | HR | 95% CI | *p* value | HR | 95% CI | *p* value |
| ***Clinical characteristics*** |  |  |  |  |  |  |
| Age | 1.06 | 1.05-1.08 | <0.001 | 1.06 | 1.04-1.07 | <0.001 |
| Male | 0.73 | 0.56-0.96 | 0.022 |  |  |  |
| BMI | 1 | 0.96-1.04 | 0.953 |  |  |  |
| Diabetes mellitus | 1.31 | 1.02-1.69 | 0.038 |  |  |  |
| Hypertension | 1.48 | 1.12-1.95 | 0.005 |  |  |  |
| Hyperlipidemia | 0.99 | 0.76-1.28 | 0.933 |  |  |  |
| Smoking | 0.83 | 0.65-1.06 | 0.129 |  |  |  |
| Prior PCI | 1.71 | 1.32-2.21 | <0.001 | 1.61 | 1.24-2.09 | <0.001 |
| Prior CABG | 1.96 | 1.23-3.13 | 0.005 |  |  |  |
| Prior MI | 1.61 | 1.19-2.19 | 0.002 |  |  |  |
| Prior cerebrovascular disease | 2.07 | 1.53-2.81 | <0.001 | 1.57 | 1.16-2.13 | 0.005 |
| Peripheral vascular disease | 1.45 | 0.77-2.72 | 0.252 |  |  |  |
| Chronic obstructive pulmonary disease | 2.25 | 1.29-3.93 | 0.004 |  |  |  |
| STEMI | 1.21 | 0.92-1.59 | 0.177 |  |  |  |
| NSTEMI | 0.89 | 0.54-1.47 | 0.644 |  |  |  |
| UA | 0.88 | 0.68-1.14 | 0.323 |  |  |  |
| Left ventricular dysfunction | 2.02 | 1.34-3.02 | 0.001 | 1.79 | 1.19-2.69 | 0.005 |
| Renal insufficiency | 7.34 | 1.03-52.34 | 0.047 |  |  |  |
| ***Procedural characteristics*** |  |  |  |  |  |  |
| Chronic total occlusion | 0.94 | 0.57-1.56 | 0.813 |  |  |  |
| Left main artery disease | 1.44 | 0.92-2.25 | 0.107 |  |  |  |
| Moderate to severe calcification | 1.58 | 1.18-2.13 | 0.002 |  |  |  |
| Lesion length | 1 | 1-1.01 | 0.606 |  |  |  |
| Lesion number | 1.32 | 1.13-1.54 | 0.001 | 1.34 | 1.15-1.57 | <0.001 |
| Minimum lesion diameter | 0.59 | 0.38-0.94 | 0.025 |  |  |  |
| Drug-eluting stent | 0.65 | 0.4-1.07 | 0.089 |  |  |  |
| ***Laboratory test*** |  |  |  |  |  |  |
| AST, IU/L | 1 | 1-1 | <0.001 |  |  |  |
| ALT, IU/L | 1 | 1-1 | 0.785 |  |  |  |
| Albumin, g/dL | 0.95 | 0.92-0.98 | <0.001 |  |  |  |
| Total bilirubin, umol/L | 0.98 | 0.96-1.01 | 0.141 |  |  |  |
| Triglycerides, mmol/L | 0.84 | 0.72-0.97 | 0.017 |  |  |  |
| Total cholesterol, mmol/L | 0.92 | 0.82-1.04 | 0.175 |  |  |  |
| LDL-C, mmol/L | 0.91 | 0.79-1.05 | 0.194 |  |  |  |
| HDL-C, mmol/L | 1.46 | 0.96-2.21 | 0.077 |  |  |  |
| Blood glucose, mmol/L | 1.06 | 1.02-1.11 | 0.002 |  |  |  |
| High-sensitivity C-reactive protein, mg/L | 1.02 | 0.99-1.05 | 0.196 |  |  |  |

BMI, body mass index; PCI, percutaneous coronary intervention; CABG, coronary artery bypass grafting; MI, myocardial infarction; STEMI, ST-segment elevation myocardial infarction; NSTEMI, non-ST-segment elevation myocardial infarction UA, unstable angina; ALT, alanine aminotransferase; AST, aspartate aminotransferase; LDL-C, low-density lipoprotein cholesterol; HDL-C, high-density lipoprotein cholesterol;

**Supplementary Table 2. Baseline characteristics according to the absence and presence of MACCE.**

|  | Non-MACCE  (n=6227) | | MACCE  (n=159) | *p* Value | |  |
| --- | --- | --- | --- | --- | --- | --- |
| ***Clinical characteristics*** | |  |  | |  | |
| Age | | 58.3 (10.3) | 64.5 (10.9) | | <0.001 | |
| Male | | 4666 (76.2) | 181 (69.9) | | 0.025 | |
| BMI | | 25.9 (3.3) | 25.9 (3.1) | | 0.932 | |
| Diabetes mellitus | | 1806 (29.5) | 92 (35.5) | | 0.044 | |
| Hypertension | | 3936 (64.2) | 189 (73.0) | | 0.005 | |
| Hyperlipidemia | | 4092 (66.8) | 173 (66.8) | | 1 | |
| Smoking | | 3587 (58.5) | 139 (53.7) | | 0.135 | |
| Prior PCI | | 1412 (23.0) | 89 (34.4) | | <0.001 | |
| Prior CABG | | 234 (3.8) | 19 (7.3) | | 0.007 | |
| Prior MI | | 813 (13.3) | 52 (20.5) | | 0.002 | |
| Prior cerebrovascular disease | | 666 (10.9) | 53 (20.5) | | <0.001 | |
| Peripheral vascular disease | | 164 (2.7) | 10 (3.9) | | 0.341 | |
| STEMI | | 1405 (22.9) | 69 (26.6) | | 0.189 | |
| NSTEMI | | 433 (7.1) | 16 (6.2) | | 0.671 | |
| UA | | 4289 (70.0) | 174 (67.2) | | 0.368 | |
| Left ventricular dysfunction | | 101 (1.7) | 11 (4.3) | | 0.004 | |
| Renal insufficiency | | 3 (0.0) | 1 (0.4) | | 0.392 | |
| ***Procedural characteristics*** | |  |  | |  | |
| Chronic total occlusion | | 406 (6.6) | 16 (6.2) | | 0.875 | |
| Left main artery disease | | 354 (5.8) | 21 (8.1) | | 0.153 | |
| Moderate to severe calcification | | 907 (14.8) | 56 (21.6) | | 0.004 | |
| Lesion number | | 28.1 (18.6) | 28.7 (18.6) | | 0.592 | |
| Lesion length | | 1.4 (0.6) | 1.5 (0.9) | | <0.001 | |
| Minimum lesion diameter | | 0.4 (0.5) | 0.3 (0.3) | | 0.069 | |
| Drug-eluting stent | | 5856 (95.6) | 242 (93.4) | | 0.141 | |
| ***Laboratory tests*** | |  |  | |  | |
| AST, IU/L | | 26.1 (31.9) | 34.7 (65.6) | | <0.001 | |
| ALT, IU/L | | 34.5 (35.3) | 33.7 (38.1) | | 0.723 | |
| Albumin, g/dL | | 42.6 (4.2) | 41.6 (4.3) | | <0.001 | |
| Total bilirubin, μmol/L | | 14.7 (5.7) | 14.1 (5.7) | | 0.158 | |
| Triglycerides, mmol/L | | 1.8 (1.1) | 1.6 (0.8) | | 0.016 | |
| Total cholesterol, mmol/L | | 4.2 (1.1) | 4.1 (0.9) | | 0.207 | |
| LDL-C, mmol/L | | 2.5 (0.9) | 2.4 (0.8) | | 0.19 | |
| HDL-C, mmol/L | | 1.0 (0.3) | 1.1 (0.3) | | 0.078 | |
| Blood glucose, mmol/L | | 6.4 (2.4) | 6.8 (3.0) | | 0.002 | |
| High-sensitivity C-reactive protein, mg/L | | 3.6 (4.1) | 4.0 (4.2) | | 0.166 | |
| ***Liver fibrosis scores*** | |  |  | |  | |
| APRI | | 0.3 (0.6) | 0.4 (0.8) | | 0.004 | |
| AST/ALT ratio | | 0.9 (0.8) | 1.3 (1.6) | | <0.001 | |
| Forns score | | 5.7 (1.3) | 6.0 (1.2) | | <0.001 | |
| NFS | | -1.4 (1.4) | -0.6 (2.0) | | <0.001 | |

Variables are shown as mean (SD) or n (%). MACCE, major adverse cardiac and cerebrovascular events. BMI, body mass index; PCI, percutaneous coronary intervention; CABG, coronary artery bypass grafting; MI, myocardial infarction; STEMI, ST-segment elevation myocardial infarction; NSTEMI, non-ST-segment elevation myocardial infarction UA, unstable angina; ALT, alanine aminotransferase; AST, aspartate aminotransferase; LDL-C, low-density lipoprotein cholesterol; HDL-C, high-density lipoprotein cholesterol; APRI, aspartate aminotransferase to platelet ratio index; AST/ALT ratio, aspartate aminotransferase to alanine aminotransferase ratio; NFS, nonalcoholic fatty liver disease fibrosis score.
